# Identification of recurrent combinatorial patterns of chromatin modifications at promoters across various tissue types

**DOI:** 10.1186/s12859-016-1346-5

**Published:** 2016-12-23

**Authors:** Nan Meng, Raghu Machiraju, Kun Huang

**Affiliations:** 1 Department of Computer Science and Engineering, The Ohio State University, Columbus, OH 43210 USA; 2 Department of Biomedical Informatics, The Ohio State University, Columbus, OH 43210 USA

## Abstract

**Background:**

Identification and analysis of recurrent combinatorial patterns of multiple chromatin modifications provide invaluable information for understanding epigenetic regulations. Furthermore, as more data becomes available, it is computationally expensive and unnecessary to study combinatorial patterns of all modifications.

**Methods:**

A novel framework is proposed to investigate recurrent combinatorial patterns of a subset of quantitatively selected chromatin modifications. The framework is based on heirarchical clustering and selects subsets of chromatin modifications that form distinct recurrent patterns at regulatory regions. The identified recurrent combinatorial patterns can be further utilized to discover novel regulatory regions. Data is in the form of genome wide maps of histone acetylations, methylations, and histone variant of human skeletal muscular and B-lymphocyte cells both derived from the ENCODE project.

**Results:**

A case study conducted at promoter regions is presented: four out of twelve chromatin modifications were selected, eight different promoter states were identified and the identified patterns of active promoters were further utilized to discover novel promoter regions. Several previously un-annotated promoters were discovered, further investigations confirm their promoter functions.

**Conclusions:**

This framework is approproiately general and could lead to better understanding of epigenetic regulations by discovering previously unknown regulatory regions.

**Electronic supplementary material:**

The online version of this article (doi:10.1186/s12859-016-1346-5) contains supplementary material, which is available to authorized users.

## Background

Distributions of chromatin modifications on the human genome are hardly random. As certain patterns frequently recur, it has been shown that recurrent patterns of chromatin modifications can be utilized to infer the epigenetic regulatory functions of their residing regions [1–5]. Hence, much attention has been spent on investigating recurrent patterns of chromatin modifications [1, 2, 6–17]. In particular, as the number of discovered modifications increases, current analyses are constrained by data availability. Working with the whole map of all chromatin modifications is challenging and possibly unnecessary. Instead, we propose to analyze a quantitatively selected subset of chromatin modifications. It could simplify the analysis and provide guidance for future experimental design at the same time.

Currently, there are several types of known regulatory regions and it remains an active field of research to study their regulatory mechanisms [3–6, 11, 12, 14, 18–28]. Progress has been made as more data becomes available and more algorithms are developed. For instance, many efforts were spent on analyzing chromatin modifications of in human CD4+ T cells [29, 30]. ChromSig was developed by Hon et al. to utilize combination of 21 chromatin modifications to search for commonly recurring chromatin signatures using the updated data set [3, 27]. Subsequently, ChromHMM was developed to annotate the human genome using 41 chromatin modifications by Ernst et al. [2]. The same group later annotated the human genome by 15 chromatin states based on 10 chromatin modifications [26]. It is noteworthy that computationally sophisticated methods become crucial to analyze patterns of chromatin modifications as more data becomes available. Furthermore, it also demonstrates that chromatin modifications do not contribute equally to the process of identifying recurrent patterns; which is the reason why the authors achieved decent accuracy by omitting more than three quarters of available chromatin modifications in their later study. Recently, Ernst et al. reported a new study that detects chromatin states in 127 reference epigenomes [31]. This analysis was based on approximation of multiple chromatin modifications by data imputation. Instead of using data imputation to overcome the unavailability of certain data sets, we aim to quantitatively identify a subset of available chromatin modifications. Moreover, it could also provide guideline for future experimental design on choosing chromatin modifications.

In this study, a computational framework is designed to select subsets of chromatin modifications that form distinct recurrent patterns at regulatory regions. The identified recurrent combinatorial patterns can be further utilized to discover novel regulatory regions. A case study of promoters yields encouraging results: 4 out of 12 available chromatin modifications were selected and eight different recurrent patterns were indentified. In-depth analyses show that the combinatorial patterns are associated with different states of promoters, confirmed by the expression levels of genes and enriched distributions of PolII. Recurrent combinatorial patterns of active promoters were further utilized to discover novel promoters. The identified putative promoters are shown to be related to transcription activation. Furthermore, this framework can be easily adapted to study other regulatory regions or extended to annotate the whole genome.

## Methods

### Workflow

**Fig. 1:**
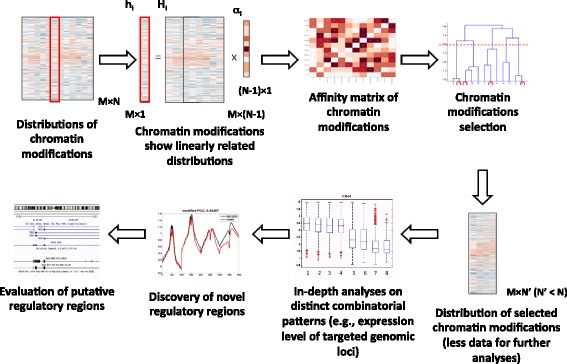
Workflow of the framework. The distribution of each chromatin modification is expressed as a weighted sum of all other modifications. The resulting coefficients are recorded in an affinity matrix. The affinity matrix is enforced to be sparse. Consequently, the chromatin modifications are clustered into different groups via hierarchical clustering. Then, a representative is selected for each cluster. After the subset is identified, the regulatory functions associated with these combinatorial patterns are further analyzed. The identified patterns then further lead to discovery of novel regulatory regions

The workflow of proposed framework is shown in Fig. 1. Firstly, data of all candidate chromatin modifications are pre-processed. Then, the distribution of each chromatin modification is expressed as a weighted sum of all other modifications. The resulting coefficients are recorded in an affinity matrix. This affinity matrix is enforced to be sparse, as the distribution of each chromatin modification is expected to be a weighted sum of few others. Consequently, the chromatin modifications are clustered into different groups via hierarchical clustering. In this step, chromatin modifications with closely related distributions are clustered into the same cluster. Then, a representative is selected from each cluster. After the subset that contains all representatives is identified, the regulatory functions associated with these combinatorial patterns are further confirmed by evidence from other databases. The identified patterns then further lead to discovery of novel regulatory regions.

### Case study at promoter region: data collection and pre-processing

Genome wide maps of two histone acetylations, eight methylations, a histone variant H2A.Z and CTCF of human skeletal muscular cells and B-lymphocyte cells were generated by the ENCODE project. For each chromatin modification, the raw data of summary tag counts obtained at every 100 bp was pre-processed before analyses.

Distributions of chromatin modifications at the -5 k to +5 k base pair (bp) region of each annotated Transcription Start Site (TSS) were extracted. The TSS list was downloaded from UCSC Genome Browser website. Overall, there are 41,413 annotated TSS from refGene. In this study, the distribution of each chromatin modification at every captured promoter region is represented by a vector of length 100 (the locus is of length 10kbp and each genomic window is of length 100 bp). Consequently, for each chromatin modification, the data matrix is of size 41,413 × 100.

### Problem formulation

Suppose distributions of *N* chromatin modifications at *M* loci are collected via ChIP-seq experiments. We separate the genome into *M* bins of size *L* and denote the vector **x**_i,j_ as

$$ {\mathbf{x}}_{i,j}=\left[\begin{array}{c}\hfill {\mathrm{x}}_{i,j,1}\hfill \\ {}\hfill {\mathrm{x}}_{i,j,2}\hfill \\ {}\hfill \vdots \hfill \\ {}\hfill {\mathrm{x}}_{i,j,L}\hfill \end{array}\right],\ i=1,2,\dots,\ N\  and\ j=1,2,\dots, M. $$

where **x**_*i*,*j*,*k*_ is the read counts for the *i* ^th^ chromatin modification at the *k* ^th^ base pair of the *j* ^th^ bin on the genome. Then the data set **H** could be written as following,

$$ \mathbf{H}=\left[{\mathbf{h}}_1\ {\mathbf{h}}_2 \dots {\mathbf{h}}_N\right]=\left(\begin{array}{ccc}\hfill {\mathbf{x}}_{1,1\ }\hfill & \hfill \cdots \hfill & \hfill {\mathbf{x}}_{N,1}\hfill \\ {}\hfill \vdots \hfill & \hfill \ddots \hfill & \hfill \vdots \hfill \\ {}\hfill {\mathbf{x}}_{1,M}\hfill & \hfill \cdots \hfill & \hfill {\mathbf{x}}_{N,M}\hfill \end{array}\right),\mathrm{where}\ {\mathbf{h}}_i=\left[\begin{array}{c}\hfill {\mathbf{x}}_{i,1}\hfill \\ {}\hfill {\mathbf{x}}_{i,2}\hfill \\ {}\hfill \vdots \hfill \\ {}\hfill {\mathbf{x}}_{i,M}\hfill \end{array}\right]. $$

### Affinity matrix of chromatin modifications

Following formulation is proposed to identify subsets of chromatin modifications forming recurrent patterns on the genome. Suppose there exists a subspace **P** that few chromatin modifications reside. Then the distribution of one chromatin modification could be expressed by linear sum of distributions of remaining chromatin modifications in the same subspace, as follows

$$ {\mathbf{h}}_i={\displaystyle \sum_{j\ne i}^{j\in \mathbf{P}}}{\mathbf{h}}_j{\alpha}_j,\  or\ {\boldsymbol{h}}_i={\displaystyle \sum_{\alpha_j=0,\ j\ne i}^{j\notin \boldsymbol{P}}}{\boldsymbol{h}}_j{\alpha}_j, $$

where α_*j*_ = 0 for all *j* ∈ **P**. Here α_j_ could be considered as a coefficient measuring how the two distributions of *i* ^th^ and *j* ^th^ chromatin modifications related. Furthermore, this could be rewritten as **h**_*i*_ = **Hα**_*i*_, where α_*ii*_ = 0 and α_*i*_∈R^*N*^ and |α_*i*_|_0_ = |P|-1. This formulation follows the assumption that a distribution can be explained by the closely related distributions of other chromatin modifications. Hence, to calculate α_*i*_, it shall follow, **min** ‖**α**_*i*_‖_0_ s. t. **h**_*i*_ = **Hα**_*i*_, α_*ii*_ = 0.

As functions in *L*_0_ space is non-convex, here the formulation is relaxed to minimize the tightest convex relaxation of the *L*_0_-norm, ie **min** ‖**α**_*i*_‖_1_ s. t. **h**_*i*_ = **Hα**_*i*_, α_*ii*_ = 0, which can be solved efficiently and prefers sparse solutions. This sparse optimization program could also be rewritten for all data points *i* = 1, …, N in matrix form as

$$ \mathbf{min}\left\Vert \mathbf{A}\right\Vert {}_1\ \mathrm{s}.\mathrm{t}.\kern0.5em \mathbf{H}=\mathbf{H}\mathbf{A},\ \mathrm{diag}\left(\mathbf{A}\right)=0, $$

where **A** ∈R^*N×N*^. This affinity matrix **A** is then used to cluster chromatin modifications. This formulation is inspired by Sparse Subspace Clustering [32].

### Selection of chromatin modifications and identification of combinatorial patterns

The affinity matrix A is then utilized to cluster chromatin modifications via hierarchical clustering. Each cluster is considered as a collection of chromatin modifications displaying linearly related distributions. Consequently, one chromatin modification is selected to represent the distribution signal of each cluster. After the representative subset is selected, distributions of all selected modifications are concatenated as one vector. Recurrent combinatorial distribution patterns are then identified by the *K*-means clustering. Here, it is hypothesized that recurrent combinatorial patterns are indicators of different states of regulatory regions. Hence, each pattern is further analyzed to confirm if they are indeed associated with epigenetic regulatory functions.

### Discovery of novel regulatory regions

The identified combinatorial patterns are then utilized to discover novel regulatory regions. Here, Pearson correlation coefficient (PCC) is used to quantify the similarity between distributions of two chromatin modifications. The similarity metric is defined as the mean of correlation coefficients of each pair of chromatin modifications. Putative regulatory regions are selected by thresholding the similarity metrics. The quality of the putative regulatory regions is further analyzed by confirming with existing annotations of the human genome and other data evidence.

In this study, ToppGene was used to study the enriched biological functions of gene groups displaying identified combinatorial patterns at promoter regions. Putative promoters are further analyzed by using evidence from other databases. Other approaches to examine the putative promoters include the investigation of the expression levels of downstream regions and PolII distributions, which are usually considered as good indicators of promoter activities.

## Results

### Subset identification

Data from human skeletal muscular cells (HSMM) and B-lymphocyte cells (GM12878) were used in this study. Overall, this study includes twelve chromatin modifications: two histone acetylations, eight histone methylations, one histone variant H2A.Z and transcriptional repressor CTCF. Annotation of promoters was obtained from UCSC Genome Browser refGene annotation.

Affinity matrix of chromatin modifications was generated for each cell line individually (see Methods section). Here, the hypothesis is that the distribution of one chromatin modification mark could be expressed as a weighted sum of few related others. Therefore, the resulting affinity matrix shall be sparse. To further enforce this assumption, the value of parameter λ is empirically tested and selected.

#### Value of λ was chosen by empirical tests

**Fig. 2:**
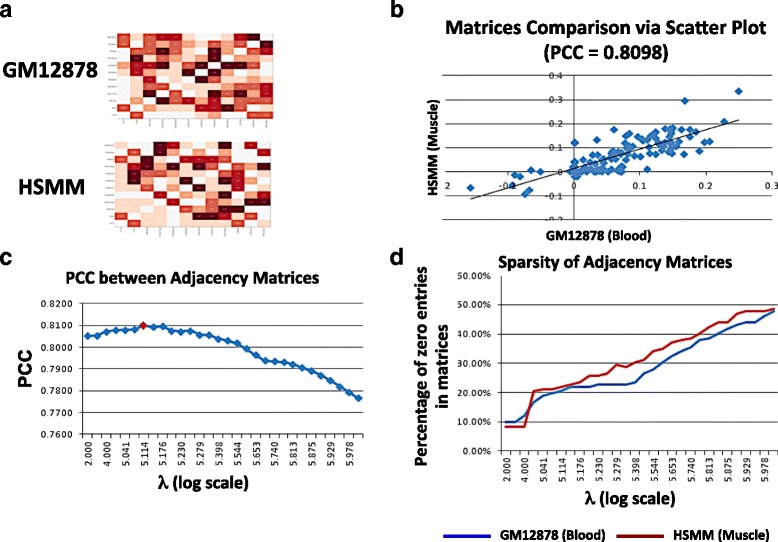
Value of λ is empirically selected by comparing the two affinity matrices generated based on data from two different cell lines. **a** Heatmaps of the affinity matrices (12 chromatin modifications) for datasets of GM12878 and HSMM cell lines. There are 66 pairs of chromatin modifications. **b** The affinity values are plotted in the scatter plot to compare the 66 pairs of chromatin modifications. The X coordinate is from cell line GM12878, the Y coordinate is from cell line HSMM. If the affinities between chromatin modifications are close, the correlation (PCC) between X and Y axis should be relatively high. **c** Changes of PCC based on different values of λ. The λ that associates with the highest PCC is then used (λ = 1.3E5). **d** As the value of λ increases, the sparsity of the two affinity matrices also increases

Since the value of λ has great impact on the sparsity of the resulting affinity matrix, it was empirically chosen by comparing two affinity matrices. Previous studies show that recurrent patterns at promoter regions remain cell type invariant [12, 25]. Hence, the affinity matrices from the two cell lines shall remain similar to each other. To compare the similarity between the two affinity matrices, the PCC between all matching entries were calculated based on different choice of λ. The value of λ that gives the highest PCC was chosen, as shown in Fig. 2.

#### Clustering chromatin modifications

**Fig. 3:**
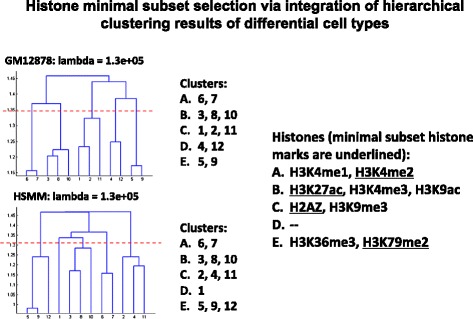
Hierarchical clustering and subset selection of chromatin modifications. The resulting clusters from both cell lines are highly overlapped (denoted by the index number of each chromatin modifications, the bold numbers indicate overlapping clusters). One chromatin modification is selected as the representative of each cluster. The chromatin modifications selected in this study are underlined

To divide the set of chromatin modifications into clusters, hierarchical clustering was applied to the affinity matrices. The clustering was tested with *K* = 3,4,5 to partition a set of 12 chromatin modifications. In the end, we selected *K* = 4 by comparing the overlaps between the clusters from the two datasets. The identified chromatin modification clusters largely overlap between the two cell lines (Fig. 3). For each cluster, one chromatin modification is selected to represent the cluster. Therefore, a group of four chromatin modifications are selected to represent the overall distributions of all chromatin modifications. The selected chromatin modifications are underlined in right of Fig. 3.

### Identification of combinatorial patterns of chromatin modifications

**Fig. 4:**
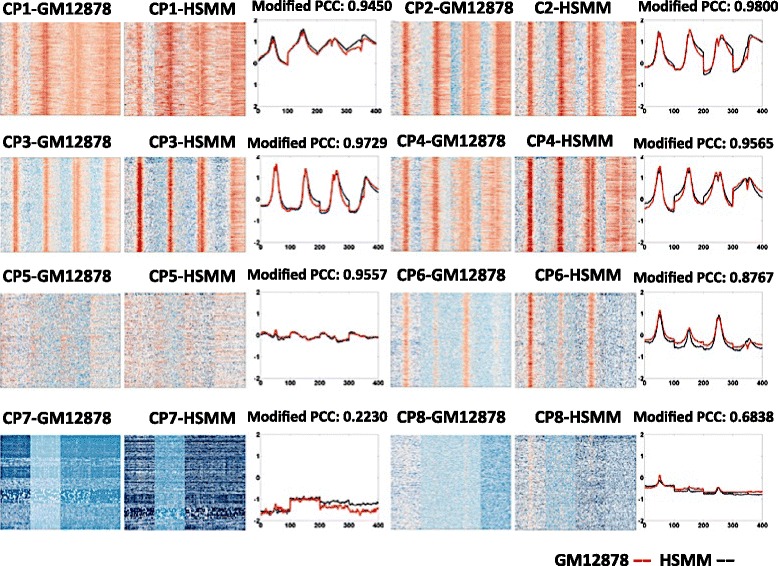
Identified combinatorial patterns (CP) and the average profile of each CP. Between the two cell lines, the patterns are similar and have high correlation. Here the modified PCC is calculated as the average of the four PCCs of the four corresponding chromatin modifications

Recurrent combinatorial patterns of chromatin modifications were detected in both cell lines via *K*-means clustering. Firstly, the distributions of selected chromatin modifications are concatenated as one vector. Therefore, for each known promoter, a vector of length N’ × L is generated to represent the combinatorial distribution. Then, the *K*-means clustering was performed to identify recurrent combinatorial patterns at promoters. To select an optimal value of *K*, the silhouette values and sum of point-to-centroid distances were examined for *K* value varies from 2 to 20. *K* is set to 8 for both cell lines (Table 1 shows the sizes of all clusters in both cell lines) as the silhouette values are high, sum of point-to-centroid distances are low and the patterns show clear visual differences. Figure 4 shows the clustering results from both cell lines. The recurrent combinatorial patterns (CP) are ranked by the expression level of their target genes. It is observed that there exist similar combinatorial patterns in both cell lines. Similarity between two combinatorial patterns is calculated by modified PCC: the mean of PCC among all matching pairs of chromatin modifications. As shown in Fig. 4 and Table 1, modified PCCs between combinatorial patterns discovered in both cell lines are quite high.

**Table 1:** Sizes of identified clusters and the correlations between matching clusters from the two cell lines

Cluster Sizes	GM12878	HSMM	Modified PCC
CP1	4655	3190	0.945
CP2	6954	6486	0.980
CP3	6154	7551	0.973
CP4	4145	4572	0.956
CP5	2799	3657	0.956
CP6	5259	6865	0.877
CP7	795	995	0.223
CP8	10652	8097	0.684

**Fig. 5:**
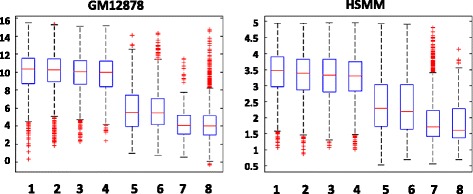
Expression levels of identified clusters from the two cell lines. It is clear that CP 1–4 have higher expression levels in both cell lines than CP 5–6 (corresponding to weak promoters) and CP7-8 (corresponding to poised promoters)

Analyses of expression levels of genes show different combinatorial patterns are associated with different promoter states. Each state is considered to carry out a different epigenetic regulatory function. It is observed that the same recurrent combinatorial pattern is associated with similar expression levels in both cell lines. As Fig. 5 shows, the combinatorial patterns could be divided into three groups: patterns of active promoters (CP1-CP4), weak promoters (CP5, CP6) and inactive/poised promoters (CP7, CP8).

**Fig. 6:**
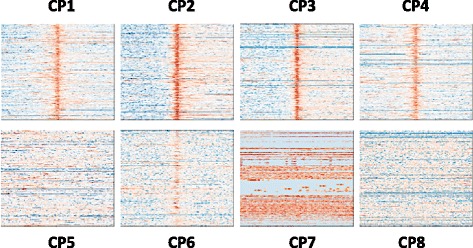
Distributions of PolII for identified clusters. For CP1-4, the PolII distribution levels are very high, comparing to that at the CP5-8 loci on genome

Another indicator of activation of transcription is the enriched distribution of PolII at promoters, as it is the enzyme that catalyzes the transcription at TSS. Here, distributions of PolII at promoter regions of genes were investigated as well. As plotted in Fig. 6, results show that there is significant PolII enrichment at active promoters (CP1-CP4), and scarce distribution on weak promoters (CP5, CP6) and almost no clear distribution at poise promoters (CP7, CP8).

**Fig. 7:**
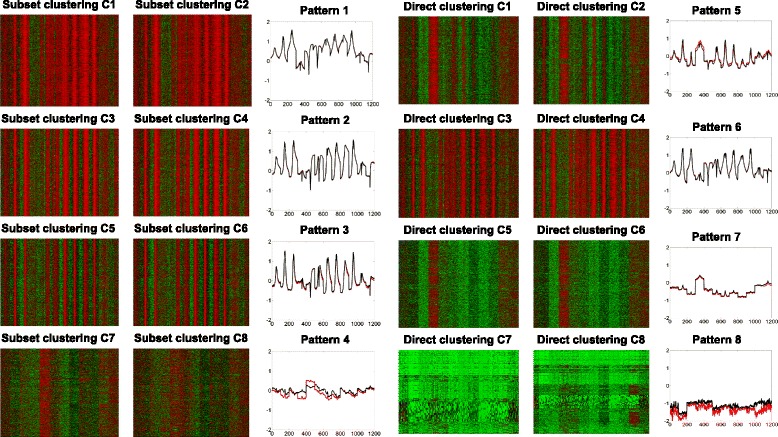
Recurrent patterns from clustering selected subset (left columns of heatmaps) of chromatin modifications and the full set (right columns of heatmaps). The average pattern profiles detected based on subset (*red*) and full set (*black*) of chromatin modifications are also plotted. While the clusters on the left columns (columns 1 and 4) are generated by the four modifications, the profiles for all 12 modifications are still shown in the heatmaps

**Fig. 8:**
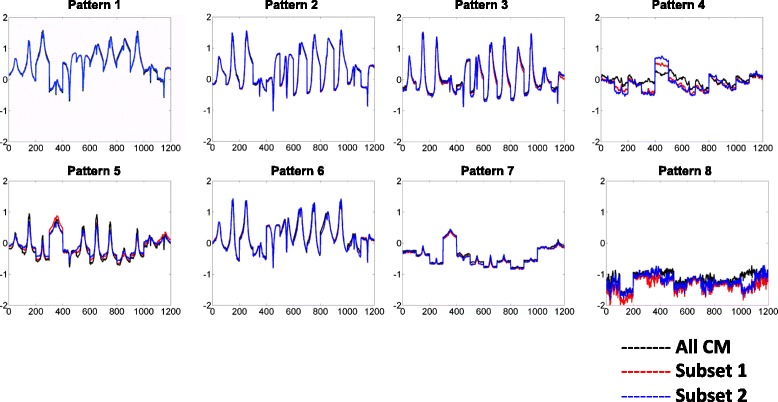
By the framework proposed, we could also select another subset of chromatin modifications and achieve similar results. Here, *black* line denotes the pattern profiles detected by clustering full set of chromatin modifications; *red* line is for the patterns detected by using H3K4me2, H3K27ac, H2Az and H3K79me2; *blue* line is for patterns detected by using H3K4me1, H3K9as, H3K9me3, and H3K36me3

**Fig. 9:**
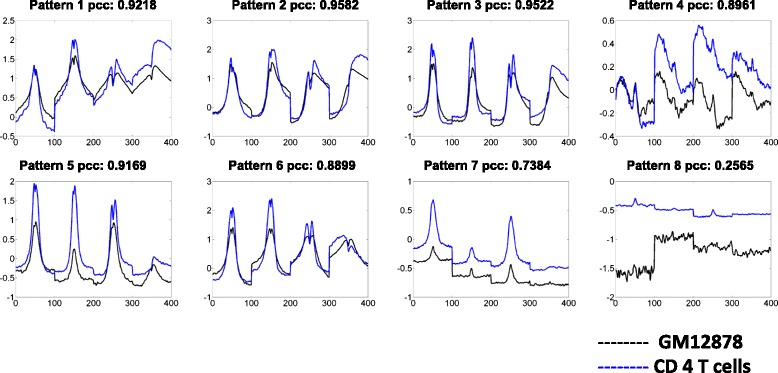
Test the pipeline in different dataset. Similar patterns are also detected by performing clustering on the same subset of chromatin modifications (H3K4me2, H3K27ac, H2Az and H3K79me2) in GM12878 (*black*) and CD4 T (*blue*) cells

To further evaluate the selected subsets of chromatin modifications, we compared the clusters identified by clustering all available chromatin modifications and the selected subset, as shown in Fig. 7. Our experiment shows that the recurrent patterns recovered by performing clustering on the two data sets are quite similar. Hence, our selected subset of chromatin modification simplified the identification of recurrent patterns without compromising accuracy. Moreover, we also selected another subset of chromatin modifications (H3K4me1, H3K9ac, H3K9me3, and H3K36me3) from Fig. 3. Our experiment shows that the recurrent patterns recovered by the two subsets are quite similar as well, as shown in Fig. 8. Based on the original subset (H3K4me2, H3K27ac, H2Az and H3K79me2), similar recurrent patterns were also detected in CD 4 T cells, as shown in Fig. 9.

### Distinct combinatorial patterns are indicators of specific regulatory functions

To thoroughly investigate the differences among genes associated with patterns of active promoters, they are further examined with functional enrichment analyses. Results show that genes displaying CP1 are enriched with tissue specific functions and genes displaying CP2-4 are associated with mostly housekeeping functions.

#### CP1 (tissue specific genes)

Functional enrichment analysis of genes displaying CP1 at promoter regions yield several tissue specific biological processes and mouse phenotypes. The enriched GO terms and associated *p*-values are listed in Table 2 (with details in Additional file 1: Table S1).

**Table 2:** Top enriched GO terms for genes with CP1 at promoters (details in Additional file 1: Table S1)

GM12878		HSMM	
*CP1: Biological Process*			
Lymphocyte activation	4.06E-12	cardiovascular system development	8.08E-22
Leukocyte activation	2.05E-11	muscle structure development	2.31E-21
Immune response	5.48E-11	skeletal system development	8.68E-19
*CP1: Mouse Phenotype*			
Abnormal leukocyte physiology	3.12E-26	abnormal axial skeleton morphology	4.27E-10
Abnormal lymphocyte physiology	5.95E-26	abnormal muscle morphology	1.69E-09
Abnormal hematopoietic system physiology	9.38E-26	abnormal thoracic cage morphology	3.81E-09

#### CP2-4 (housekeeping genes)

For genes that displaying CP2, CP3 and CP4 at promoter regions, functional enrichment analyses indicate that they are mostly associated with housekeeping functions. It is noteworthy that the enriched functions usually overlap significantly for genes displaying the same pattern from both cell lines. The enrichment analyses results are listed in Table 3 (with details in Additional file 2: Table S2, Additional file 3: Table S3, and Additional file 4: Table S4 for CP2, CP3, and CP4 respectively). GO terms that are enriched in gene groups from both cell lines are listed in bold. The remaining non-overlapping GO term are mostly related to the overlapping GO terms. For example, in Table 3 for CP2, one GO term enriched in both cell lines is “regulation of cellular protein metabolic process”, and the non-overlapping GO terms include “negative regulation of metabolic process” and “negative regulation of cellular metabolic process”. Even though some GO terms do not appear in both columns, the functions of both gene groups are closely related.

**Table 3:** Riched GO BP terms for genes with CP2to CP4 at promoters (details in Additional file 2: Table S2, Additional file 3: Tables S3, Additional file 4: Tables S4)

GM12878		HSMM	
***CP2-Biological Process***			
** Cell cycle**	2.87E-36	**regulation of cellular protein metabolic process**	1.07E-40
** Mitotic cell cycle**	1.04E-34	**negative regulation of macromolecule metabolic process**	1.62E-37
** Single-organism organelle organization**	1.37E-29	**cell cycle**	2.17E-33
***CP3-Biological Process***			
** tRNA metabolic process**	2.71E-11	**protein modification by small protein conjugation or removal**	1.15E-14
** ncRNA metabolic process**	3.64E-09	**protein modification by small protein conjugation**	2.62E-13
** tRNA processing**	6.71E-09	**ncRNA metabolic process**	5.48E-13
** Protein modification by small protein conjugation or removal**	3.57E-08	cellular respiration	9.00E-13
** ncRNA processing**	1.07E-07	**tRNA metabolic process**	1.17E-12
***CP4-Biological Process***			
** RNA processing**	5.30E-09	**RNA processing**	5.51E-15
** tRNA metabolic process**	4.06E-08	**ncRNA metabolic process**	1.40E-12
** DNA metabolic process**	1.44E-06	**DNA metabolic process**	3.64E-11
tRNA processing	5.01E-06	**ncRNA processing**	4.52E-11
** Cellular response to DNA damage stimulus**	5.93E-06	**DNA repair**	1.04E-09
** ncRNA metabolic process**	6.76E-06	**cellular response to DNA damage stimulus**	1.41E-09
** ncRNA processing**	7.45E-06	RNA modification	6.34E-09

Recurrent GO that are enriched from both cell lines are listed in bold

### Discovery of novel promoters

**Fig. 10:**
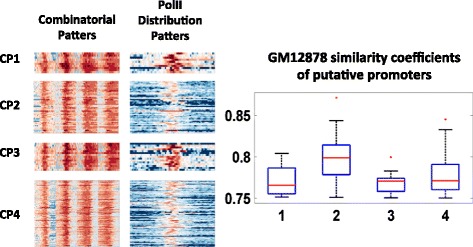
Combinatorial patterns and PolII distributions of putative promoters

As the identified recurrent combinatorial patterns associate with promoters of different states, they could be utilized to discover novel promoters. In this study, un-annotated promoter regions are discovered if they display identified patterns of active promoters. Here, the human genome is divided into 10 k bps loci with 2 k bps sliding window. The combinatorial distribution at each locus was then compared to the identified recurrent patterns of active promoters (Fig. 10). Here the similarity between two combinatorial patterns is calculated as the mean of the PCC of all matching pairs. A locus is considered as a putative promoter only if similarity coefficients of all individual PCC are above certain threshold (0.75 in this study). After all the candidate loci are selected, loci with high similarity scores are further analyzed. The search is carried out on both DNA strands.

#### Evaluation of the putative promoters

**Fig. 11:**
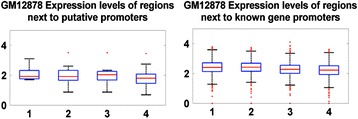
Comparison of expression levels of regions regulated by putative (left) and identified active promoters (right)

Putative promoter regions are further analyzed: the expression levels of downstream regions are examined along with the PolII distributions. Investigations show that the downstream regions from putative promoters have similar expression levels with the genes that displaying the same patterns at their promoters, as shown in Fig. 11. Furthermore, investigations also show putative promoter regions display PolII distribution patterns that are expected for active promoter regions, as shown in Fig. 7. Further analyses indicated that putative promoters mostly consist of promoter regions of non-coding RNAs, exons of known genes along gene body and regions without annotations. The breakdown of the putative promoters is listed in Table 4.

**Table 4:** Further analyses of the identified putative promoters

	Number of putative promoters	Regions overlaps with annotations		Un-annotated regions
		Regions between gene bodies	Regions within gene bodies	
CP1	10	3	3	4
CP2	46	6	17	25
CP3	15	1	4	11
CP4	105	27	8	72

**Fig. 12:**
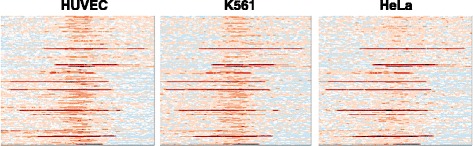
PolII distributions at putative promoters (identified in cell line GM12878) in other cell lines

As shown above, the un-annotated regions downstream of active promoter patterns also have similar expression levels of known genes with the same promoter pattern, and similar PolII distributions. The PolII distributions of putative promoters were also investigated in other cell lines, such as HUVEC, K562 and HeLa (Fig. 12). Results show that the putative promoters in these three cell lines also display enriched PolII distributions. One interesting observation is that the PolII distributions are different in these three cell lines, suggesting that some identified promoters are likely to be tissue specific. Hence, some of them are active in GM12878 but not as much in other cells.

## Discussion and conclusion

In this study, we propose a framework to investigate recurrent combinatorial patterns of chromatin modifications at regulatory regions. As certain chromatin modifications are not available for analyses, our method focuses on exploring the distinct combinational patterns of selected modifications. The framework is demonstrated in detail by a case study conducted at promoter regions. By using the proposed framework, a subset of available chromatin modifications was successfully identified based on their distribution patterns at promoter regions. Specifically, we identified four groups of chromatin modifications that provide four representative modifications. Interestingly, in the Epigenome Roadmap project, six types of chromatin modifications (H3K4me1, H3K4me3, H3K9ac, H3K9me3, H3K27me3, H3K36me3) were adopted for characterizing chromatin states [33]. Among them, H3K4me1 is in the Cluster A (Fig. 3), H3K4me3 and H3K9ac are in Cluster B, H3K9me3 is in the Cluster C, and H3K36me3 is in the Cluster E. In addition, in [31], five chromatin modifications (H3K4me1, H3K4me3, H3K9me3, H3K27me3, and H3K36me3) were adopted for imputing other chromatin marks. These five selected modifications also span the four clusters detected using our methods. These observations clearly demonstrated that the clusters we identified are comprehensive for selecting representative modifications. In addition, our method also suggested that there are relationships between the modifications within each cluster that cannot be effectively detected using traditional Pearson correlation method. For Cluster A, while H3K4me1 is known to preferentially bind to active enhancers, H3K4me2 is known to exist in both active enhancers and promoters. Thus the correlation between H3K4me1 and H3K4me2 over the neighborhood of TSS regions is not strong. Since our analysis focuses on regions within 5Kb of the TSS regions, there are complementary patterns for the promoters and the proximal enhancers for active genes that can be detected by our method. The three chromatin modifications in Cluster B are H3K27ac, H3K4me3 and H3K9ac. Interestingly, using a two step computational model, Dong et al. [34] showed that H3K4me3 has provide similar information on gene transcription as the activating marks H3K27ac and H3K9ac. In Cluster D, the two chromatin modifications H3K36me3 and H3K79me2 are both activating marks binding to gene bodies. However, H3K36me3 occurs preferentially on the 3’ of the genes while H3K79me2 is present more in the 5’ region. Thus they do not always show strong correlations. Instead the subspace model can detect the complementary relationships between them. The relationship between H2A.Z and H3K9me3 in Cluster C are less well known. H3K9me3 is known to mark heterochromatin [35]. Some recent studies showed that the H2A.Z and H3K9me3 co-localize in certain heterochromatin regions but H2A.Z have much wider presence than H3K9me3 [36, 37].

Furthermore, instead of just assigning chromatin states and predicting gene activities, we examine the distribution patterns of the four representative modifications to categorize the genes as it has been shown previously that different distribution patterns of certain chromatin modifications may be associated with different gene functions [13, 17]. Specifically, the recurrent patterns formed by the selected subset of chromatin modifications were identified. Our investigations show that the identified recurrent combinatorial patterns associated with different states of promoters, confirmed by the expression levels of downstream genes and PolII distributions at promoter regions. Importantly, our results showed that even for active genes, they have different distribution patterns for the selected modifications corresponding to different functions. The most active group contains tissue specific genes while active genes in the other groups are usually involved in more household functions such as cell cycle, RNA metabolism and protein synthesis.

In addition, the identified patterns were further utilized for discovering putative promoters. Further analysis show that the putative promoters are indeed related to activation of transcription. Promoter regions were chosen to demonstrate this framework as their targeted regions are easy to locate. It is worth mentioning that this framework can be easily adapted to other regulatory regions with suitable data sets, or extend to study genome wide recurrent patterns/annotate the whole human genome.

A major limitation of our current analysis is that we focused on the TSS regions. It has been shown that different regulatory regions may have different combinatorial patterns [1] and we plan to extend the analysis to whole genome in our future work.

In conclusion, we present a computational framework to identify relationships of chromatin modifications beyond correlation analysis and identified representative modifications that can be further used to categorize functional groups of genes as well as predicting new gene regulatory regions.

## Additional files

Additional file 1: Enriched GO terms for genes displaying CP1 at their promoters. (DOCX 14 kb)
Additional file 2: Enriched GO terms for genes displaying CP2 at their promoters. (DOCX 15 kb)
Additional file 3: Enriched GO terms for genes displaying CP3 at their promoters. (DOCX 14 kb)
Additional file 4: Enriched GO terms for genes displaying CP4 at their promoters. (DOCX 14 kb)

## Abbreviation

TSS: Transcription starting site
