# Supplementary material for: Identification of recurrent combinatorial patterns of chromatin modifications at promoters across various tissue types
Source: BMC Bioinformatics. 2016 Dec 23;17(Suppl 17):534. doi: 10.1186/s12859-016-1346-5 (PMC5259941; doi:10.1186/s12859-016-1346-5)
Supplement: Additional file 1: — Enriched GO terms for genes displaying CP1 at their promoters. (DOCX 14 kb) [file 12859_2016_1346_MOESM1_ESM.docx]

**Additional file 1: Table-S1 Enriched GO terms for genes displaying CP1 at their promoters.**

| **CP1: Biological Process** | | | | |
| --- | --- | --- | --- | --- |
| GM12878 | | HSMM | | |
| lymphocyte activation | 4.06E-12 | cardiovascular system development | | 8.08E-22 |
| leukocyte activation | 2.05E-11 | muscle structure development | | 2.31E-21 |
| immune response | 5.48E-11 | skeletal system development | | 8.68E-19 |
| regulation of lymphocyte activation | 6.79E-11 | muscle tissue development | | 1.60E-17 |
| T cell activation | 1.39E-10 | striated muscle tissue development | | 5.22E-17 |
| regulation of leukocyte activation | 2.23E-10 | muscle organ development | | 2.85E-16 |
| regulation of immune system process | 4.62E-10 | skeletal system morphogenesis | | 1.72E-15 |
| immune effector process | 8.76E-09 | embryonic skeletal system development | | 4.92E-15 |
| positive regulation of T cell activation | 1.09E-08 | heart development | | 1.04E-12 |
| regulation of T cell activation | 1.12E-08 | embryonic skeletal system morphogenesis | | 1.21E-12 |
| **CP1: Mouse Phenotype** | | | | |
| GM12878 | | HSMM | | |
| abnormal leukocyte physiology | 3.12E-26 | abnormal axial skeleton morphology | 4.27E-10 | |
| abnormal lymphocyte physiology | 5.95E-26 | abnormal muscle morphology | 1.69E-09 | |
| abnormal hematopoietic system physiology | 9.38E-26 | abnormal thoracic cage morphology | 3.81E-09 | |
| abnormal immune cell physiology | 1.52E-25 | abnormal skeleton morphology | 5.59E-08 | |
| abnormal cell-mediated immunity | 2.36E-25 | abnormal joint morphology | 7.09E-08 | |
| abnormal blood cell physiology | 4.31E-25 | abnormal cardiovascular system morphology | 7.65E-08 | |
| abnormal adaptive immunity | 4.32E-25 | abnormal cardiovascular development | 8.17E-08 | |
| abnormal T cell physiology | 3.52E-24 | abnormal rib morphology | 1.32E-07 | |
| abnormal lymphocyte morphology | 4.07E-23 | skeleton phenotype | 2.09E-07 | |
| abnormal hemopoiesis | 5.24E-22 | abnormal skeletal muscle morphology | 9.56E-07 | |
