# Supplementary material for: Identification of recurrent combinatorial patterns of chromatin modifications at promoters across various tissue types
Source: BMC Bioinformatics. 2016 Dec 23;17(Suppl 17):534. doi: 10.1186/s12859-016-1346-5 (PMC5259941; doi:10.1186/s12859-016-1346-5)
Supplement: Additional file 2: — Enriched GO terms for genes displaying CP2 at their promoters. (DOCX 15 kb) [file 12859_2016_1346_MOESM2_ESM.docx]

**Additional file 2: Table-2 Enriched GO terms for genes displaying CP2 at their promoters.**

| **CP2-Biological Process** | |  | |  |  | |
| --- | --- | --- | --- | --- | --- | --- |
| GM12878 | | | HSMM | | | |
| **cell cycle** | 2.87E-36 | | **regulation of cellular protein metabolic process** | | | 1.07E-40 |
| **mitotic cell cycle** | 1.04E-34 | | **negative regulation of macromolecule metabolic process** | | | 1.62E-37 |
| **single-organism organelle organization** | 1.37E-29 | | regulation of cellular component organization | | | 2.40E-36 |
| **cell cycle process** | 1.75E-29 | | negative regulation of metabolic process | | | 2.26E-35 |
| mitotic cell cycle process | 5.91E-29 | | **cell cycle** | | | 2.17E-33 |
| **intracellular transport** | 3.13E-26 | | **single-organism organelle organization** | | | 6.55E-31 |
| cellular macromolecule catabolic process | 1.14E-25 | | transcription from RNA polymerase II promoter | | | 2.64E-30 |
| single-organism intracellular transport | 5.40E-25 | | **programmed cell death** | | | 2.65E-30 |
| **regulation of cellular protein metabolic process** | 7.11E-25 | | regulation of protein modification process | | | 5.82E-30 |
| viral process | 2.57E-24 | | negative regulation of cellular metabolic process | | | 5.85E-30 |
| multi-organism cellular process | 4.41E-24 | | apoptotic process | | | 9.19E-30 |
| cellular response to stress | 5.67E-24 | | cellular protein localization | | | 5.43E-29 |
| protein modification by small protein conjugation or removal | 7.06E-24 | | protein phosphorylation | | | 6.46E-29 |
| macromolecule catabolic process | 8.07E-24 | | **cellular macromolecule localization** | | | 6.69E-29 |
| **programmed cell death** | 3.53E-23 | | **mitotic cell cycle** | | | 1.52E-28 |
| **negative regulation of macromolecule metabolic process** | 6.33E-23 | | regulation of transcription from RNA polymerase II promoter | | | 2.03E-28 |
| protein localization | 9.59E-23 | | **intracellular transport** | | | 1.07E-27 |
| nucleobase-containing compound catabolic process | 1.38E-22 | | phosphorylation | | | 1.94E-27 |
| **cellular macromolecule localization** | 3.51E-22 | | enzyme linked receptor protein signaling pathway | | | 1.98E-27 |
| interspecies interaction between organisms | 4.67E-22 | | **cell cycle process** | | | 2.30E-27 |
