# Supplementary material for: Identification of recurrent combinatorial patterns of chromatin modifications at promoters across various tissue types
Source: BMC Bioinformatics. 2016 Dec 23;17(Suppl 17):534. doi: 10.1186/s12859-016-1346-5 (PMC5259941; doi:10.1186/s12859-016-1346-5)
Supplement: Additional file 3: — Enriched GO terms for genes displaying CP3 at their promoters. (DOCX 14 kb) [file 12859_2016_1346_MOESM3_ESM.docx]

**Additional file 3: Table-S3 Enriched GO terms for genes displaying CP3 at their promoters.**

| **CP3-Biological Process** | | | |
| --- | --- | --- | --- |
| GM12878 | | HSMM | |
| **tRNA metabolic process** | 2.71E-11 | **protein modification by small protein conjugation or removal** | 1.15E-14 |
| **ncRNA metabolic process** | 3.64E-09 | **protein modification by small protein conjugation** | 2.62E-13 |
| **tRNA processing** | 6.71E-09 | **ncRNA metabolic process** | 5.48E-13 |
| **protein modification by small protein conjugation or removal** | 3.57E-08 | cellular respiration | 9.00E-13 |
| **ncRNA processing** | 1.07E-07 | **tRNA metabolic process** | 1.17E-12 |
| protein polyubiquitination | 1.89E-07 | cellular response to DNA damage stimulus | 1.52E-12 |
| **electron transport chain** | 1.90E-07 | RNA processing | 5.05E-12 |
| **protein modification by small protein conjugation** | 2.24E-07 | **protein ubiquitination** | 1.62E-11 |
| respiratory electron transport chain | 2.62E-07 | mitotic cell cycle | 1.80E-10 |
| cellular respiration | 4.12E-07 | DNA repair | 2.50E-10 |
| regulation of ligase activity | 4.73E-07 | respiratory electron transport chain | 5.18E-10 |
| **mitochondrion organization** | 5.41E-07 | **ncRNA processing** | 1.31E-09 |
| regulation of ubiquitin-protein transferase activity | 1.18E-06 | **electron transport chain** | 1.62E-09 |
| negative regulation of ubiquitin-protein transferase activity | 1.49E-06 | **tRNA processing** | 2.01E-09 |
| negative regulation of ligase activity | 1.49E-06 | **mitochondrion organization** | 2.81E-09 |
| oxoacid metabolic process | 2.00E-06 | mitotic cell cycle process | 3.25E-09 |
| carboxylic acid metabolic process | 2.39E-06 | cell cycle | 4.26E-09 |
| **protein ubiquitination** | 2.66E-06 | cellular response to stress | 5.77E-09 |
| oxidation-reduction process | 2.72E-06 | cofactor metabolic process | 8.18E-09 |
| phosphatidylinositol metabolic process | 2.83E-06 | single-organism intracellular transport | 3.24E-08 |
