# Supplementary material for: Identification of recurrent combinatorial patterns of chromatin modifications at promoters across various tissue types
Source: BMC Bioinformatics. 2016 Dec 23;17(Suppl 17):534. doi: 10.1186/s12859-016-1346-5 (PMC5259941; doi:10.1186/s12859-016-1346-5)
Supplement: Additional file 4: — Enriched GO terms for genes displaying CP4 at their promoters. (DOCX 14 kb) [file 12859_2016_1346_MOESM4_ESM.docx]

**Additional file 4: Table S4 Enriched GO terms for genes displaying CP4 at their promoters.**

| **CP4-Biological Process** | |  | |  |  |
| --- | --- | --- | --- | --- | --- |
| GM12878 | | | HSMM | | |
| **RNA processing** | 5.30E-09 | | **RNA processing** | | 5.51E-15 |
| **tRNA metabolic process** | 4.06E-08 | | **ncRNA metabolic process** | | 1.40E-12 |
| **DNA metabolic process** | 1.44E-06 | | **DNA metabolic process** | | 3.64E-11 |
| tRNA processing | 5.01E-06 | | **ncRNA processing** | | 4.52E-11 |
| **cellular response to DNA damage stimulus** | 5.93E-06 | | **DNA repair** | | 1.04E-09 |
| **ncRNA metabolic process** | 6.76E-06 | | **cellular response to DNA damage stimulus** | | 1.41E-09 |
| **ncRNA processing** | 7.45E-06 | | RNA modification | | 6.34E-09 |
| iron-sulfur cluster assembly | 1.43E-05 | | **tRNA metabolic process** | | 9.22E-09 |
| metallo-sulfur cluster assembly | 1.43E-05 | | cell cycle | | 1.05E-07 |
| chromosome organization | 1.95E-05 | | ribonucleoprotein complex biogenesis | | 1.50E-07 |
| **DNA repair** | 1.95E-05 | | rRNA metabolic process | | 7.54E-07 |
| mRNA processing | 2.81E-05 | | nucleoside monophosphate metabolic process | | 1.22E-06 |
| **transcription from RNA polymerase III promoter** | 4.03E-05 | | respiratory electron transport chain | | 1.24E-06 |
| DNA recombination | 5.67E-05 | | **transcription from RNA polymerase III promoter** | | 1.31E-06 |
| cellular respiration | 7.56E-05 | | cell cycle process | | 1.33E-06 |
